# Targeted siRNA Delivery Using Cetuximab‐Conjugated Starch for Epidermal Growth Factor Receptor‐Driven Head and Neck Squamous Cell Carcinoma

**DOI:** 10.1002/smsc.202500073

**Published:** 2025-05-15

**Authors:** Chen Benafsha, Limor Cohen, Leah Shimonov, Riki Goldbart, Tamar Traitel, Eliz Amar‐Lewis, Ramesh Chintakunta, Manu Parasad, Uzi Hadad, Moshe Elkabets, Joseph Kost

**Affiliations:** ^1^ Department of Chemical Engineering Ben‐Gurion University of the Negev Beer‐Sheva 84105 Israel; ^2^ Health Professions Faculty of Health Sciences Nursing Department Recanati School for Community Ben‐Gurion University of the Negev Beer‐Sheva 84105 Israel; ^3^ Ilse Katz Institute for Nanoscale Science & Technology Ben‐Gurion University of the Negev Beer‐Sheva 84105 Israel; ^4^ Faculty of Health Sciences The Shraga Segal Department of Microbiology, Immunology, and Genetics Ben‐Gurion University of the Negev Beer‐Sheva 84105 Israel

**Keywords:** cetuximab, EGFR, gene silencing, HNSCC, quaternized starch, targeted small interfering RNA delivery

## Abstract

Small interfering RNA (siRNA) therapy holds significant potential to disrupt oncogenic signaling pathways by targeting specific messenger RNA (mRNA) sequences. However, its clinical application is limited by challenges in developing effective delivery systems. In this study, starch, a biocompatible and biodegradable natural polysaccharide, is utilized as a carrier to enhance siRNA stability and delivery efficiency. By conjugating cetuximab to quaternized starch (Q‐starch) complexed with the siRNA, an increased specificity is achieved toward cancer cells overexpressing the epidermal growth factor receptor (EGFR). This research encompasses the synthesis, characterization, and biological evaluation of these targeted complexes, which demonstrate efficient cellular uptake and on‐target knockdown in vitro. Furthermore, these complexes exhibit notable tumor‐specific accumulation, significantly enhancing the active targeting of EGFR‐overexpressing tumors in vivo. These findings highlight the potential of the complexes to accumulate in EGFR‐expressing head and neck squamous cell carcinoma, advancing the development of starch‐based delivery systems and paving the way for further diagnostic and therapeutic applications.

## Graphical abstract

A starch‐based carrier conjugated to cetuximab enables targeted siRNA delivery to epidermal growth factor receptor‐overexpressing tumors. The complexes exhibit dual passive and active tumor targeting, effective cellular uptake, and gene silencing, offering a versatile platform for RNA therapeutics in head and neck cancer.

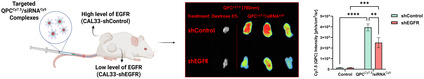

## 1 Introduction

The discovery of small interfering RNA (siRNA) mechanism has led to significant efforts to develop a versatile system to tackle various genetic diseases, particularly cancer, which is marked by diverse mutations.^[^ ^1^, ^2^ ^]^ A key benefit of siRNA therapy lies on its ability to precisely target complementary messenger RNA (mRNA) sequences, resulting in their degradation and effective inhibition of protein translation. However, developing an effective therapeutic approach is fraught with challenges, primarily due to the need for a reliable delivery system capable of transporting and releasing siRNA, specifically into cancerous cells.^[^ ^3^ ^]^ The inherent properties of siRNAs, including their size, hydrophilic nature, and negative charge, promote their rapid clearance through the renal system.^[^ ^4^ ^]^ This results in limited systemic circulation time, and reduced availability for cellular uptake in target tissues, cause to hinder their effective therapeutic use.^[^ ^5^ ^]^

One effective approach addressing these challenges is to establish a delivery system that utilizes a carrier designed to deliver the siRNA specifically to the intended target site. Various carriers have been investigated as siRNA delivery systems, including liposomes,^[^ ^6^ ^]^ polymeric nanoparticles,^[^ ^7^ ^]^ dendrimers,^[^ ^8^ ^]^ and natural polysaccharides.^[^ ^9^ ^]^ Among these, starch, a natural polysaccharide, has gained significant attention in our research group^[^ ^10^, ^11^, ^12^, ^13^ ^]^ due to its unique properties that set it apart from other carriers. Starch exhibits high biocompatibility, which helps minimize toxicity and enhances safety in biomedical applications. Unlike some synthetic carriers, which may provoke immune responses, starch is well‐tolerated by the body. Furthermore, its biodegradability ensures that it can be safely metabolized after delivering its payload, reducing the risk of long‐term accumulation in tissues or adverse effects associated with more persistent materials.^[^ ^12^, ^13^, ^14^ ^]^ Additionally, starch can be readily modified to improve its functionality, achieving higher encapsulation efficiency and stability. Its versatility allows for the attachment of specific ligands that target receptors associated with cancer cells, ensuring that the siRNA is delivered precisely where it is needed, thus maximizing therapeutic effectiveness.^[^ ^15^ ^]^

Targeted therapy, which focuses on specific tumor types and minimizes damage to healthy tissues, has revolutionized cancer treatment.^[^ ^16^ ^]^ A common overexpressed receptor, extensively studied and closely associated with the progression and malignancy of various cancers, is the epidermal growth factor receptor (EGFR).^[^ ^17^ ^]^ EGFR is a tyrosine kinase receptor that plays a crucial role in promoting cell proliferation, survival, and angiogenesis. This overexpression is commonly observed in a range of cancer types, including non‐small cell lung cancer, head and neck squamous cell carcinoma (HNSCC), and colorectal cancer, among others.^[^ ^18^ ^]^ EGFR abnormal signaling pathways lead to hyperactivation of downstream mechanisms that drive tumor growth and metastasis.^[^ ^19^, ^20^ ^]^ Cetuximab, a monoclonal antibody that specifically target and blocks EGFR activation, has shown considerable promise in cancer treatment, especially in HNSCC, by preventing downstream signaling that promotes cell proliferation and survival.^[^ ^21^, ^22^ ^]^ However, one of the challenges in utilizing cetuximab is the development of resistance, as cancer cells can activate alternative pathways that circumvent this blockade, allowing the tumor to continue its progression.^[^ ^23^, ^24^ ^]^ Despite the reported resistance observed in a broad segment of the population, the targeting antibody capability toward the target cell remains intact. Therefore, the option to utilize the cetuximab for targeting purposes, such as the focused delivery of siRNA, certainly allows for the development of an additional therapeutic tool for those resistance cases.^[^ ^25^, ^26^, ^27^ ^]^ Leveraging the tumor‐targeting capabilities of cetuximab presents an opportunity to develop a therapeutic strategy for focused delivery of RNA therapies. This approach aims to reduce systemic toxicity, minimize damage to surrounding healthy tissues, and address the well‐known challenge of hepatic clearance associated with siRNA carriers, ultimately improving the precision and efficacy of drug action.^[^ ^28^, ^29^ ^]^

In light of these findings, this study focuses on developing a specialized EGFR‐targeted siRNA therapy for HNSCC utilizing modified starch. By conjugating the cetuximab antibody to the modified starch and using its ability to actively target overexpressed EGFR HNSCC tumor cells, we aim to achieve the targeting of our delivery system for precise therapeutic outcomes. This research entails comprehensive synthesis, chemical analysis, and biological evaluation of the modified starch carrier, examining its efficacy in overcoming the barriers associated with siRNA delivery.

## 2 Results and Discussion

### 2.1 Synthesis, Chemical Analysis, and Biological Activity of Cetuximab Attached to Quaternized Starch‐PEG‐Cetuximab (QPC) Carrier

**Figure 1:**
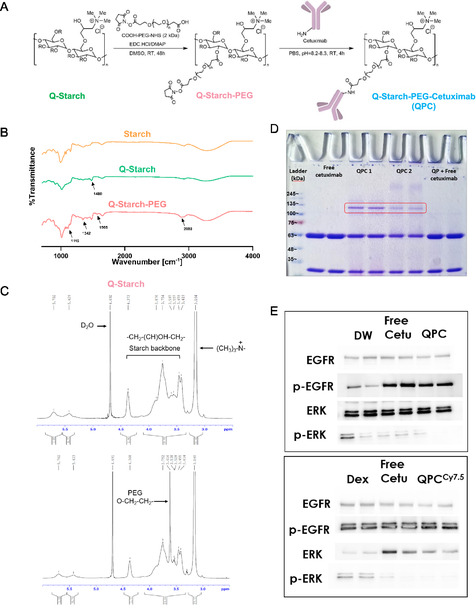
A) Illustration depicting the synthesis steps of Q‐starch‐PEG‐cetuximab (QPC). B) FTIR spectra comparison showing native starch, Q‐starch, and Q‐starch‐PEG (QP) with annotated relevant peaks. C) ^1^H NMR spectra comparison between Q‐starch and QP. D) coomassie blue SDS‐PAGE gel image confirming cetuximab attachment: lanes include free cetuximab, two QPC synthesis batches, and the mixture of QP with free cetuximab. All the samples contain total amount of 60 μg cetuximab. E) Western blot analysis demonstrates EGFR pathway inhibition by QPC and QPC^Cy7.5^. Lanes, from left include solvents (negative control), the free cetuximab (positive control), and QPC/QPC^Cy7.5^ carriers.

siRNA delivery system has been developed specifically for cells exhibiting overexpression of the EGFR. To achieve precise and effective siRNA delivery to EGFR expressing tumors, we conjugated the anti‐EGFR Ab, cetuximab, to modified starch which is extensively studied in our lab.^[^ ^11^, ^12^, ^13^ ^]^ To electrostatically complex (condense) the negatively charged siRNA to a nano‐sized delivery system, the carrier needs to have a positive charge. The positively charged carrier was generated by conjugating a quaternary‐amine group onto the starch polymer backbone to produce quaternized starch (Q‐starch). To add the targeting capability of cetuximab to the carrier, an intermediate step was introduced where a 2 kDa polyethylene glycol (PEG) chain with an NHS ester moiety at the end, is first attached to the Q‐starch, and then allow the reaction with the primary amine group in cetuximab, as illustrated in **Figure** **1**A.

The NHS‐ester, formed via EDC/NHS activation, specifically reacts with primary amines, typically found on lysine residues or the N‐terminus, ensuring efficient antibody attachment, preserving the functionality of the antigen‐binding site.^[^ ^30^ ^]^ Additionally, incorporating PEG into our carrier enhances its stability by providing steric protection against physiological proteins, thus reducing nonspecific interactions and significantly prolonging circulation time in the bloodstream.^[^ ^31^ ^]^ The substitution of the quaternary amine group was confirmed by comparing Fourier transform infrared (FTIR) spectra of native starch and Q‐starch. This analysis revealed a distinctive peak at around 1480 cm^−^^1^, indicating C—N vibrations (Figure 1B). The presence of the PEG chains is evident in the 1115 cm^−^^1^ peak, corresponding to C—O—C ether bond, 1342 and 2880 cm^−^^1^ peaks, corresponding to C—H of the —CH_2_ groups, and 1565 cm^−^^1^ corresponding to N—H vibrations of the NHS group. The observed peaks are relatively weak due to the low degree of PEG substitution that was implemented. This low degree of substitution was deliberately designed to ensure that the presence of the antibody would not interfere with the formation of the complexes. Stronger evidence for the substitution of the PEG chains is obtained from the ^1^H NMR spectra (Figure 1C), where the precise O—CH_2_—CH_2_ bonds of the PEG molecule were observed at *δ* = 3.616 compared to the Q‐starch spectra. The peak observed at *δ* = 3.164 provides further evidence of the quaternary amine modification that appears in both spectra. Verification of the chemical attachment of the cetuximab antibody to the Q‐starch‐PEG‐NHS proved to be challenging via FTIR and ^1^H NMR methods due to its low substitution. To overcome this limitation, size measurements using sulfate‐polyacrylamide gel electrophoresis (SDS‐PAGE) were employed to detect changes in cetuximab antibody size, as chemical conjugation to Q‐starch‐PEG‐NHS would result in a shift in its characteristic band size. As depicted in Figure 1D, coomassie blue SDS‐PAGE gel includes evaluation of free cetuximab, two quaternized starch‐PEG‐cetuximab (QPC) synthesis batches, and a mixture of the intermediate product, Q‐starch‐PEG (QP), mixed with free cetuximab. Discernible higher molecular weight bands are observed in the synthesized targeted QPC carrier than the heavy chain weight band of free cetuximab (marked in red rectangle). The increase in the weight of the heavy chain in the cetuximab antibody provides further evidence that conjugation with QP occurred via the lysine residues or via the N‐terminus located at the end of the heavy chains. This indicates that the critical region, the antigen‐binding site, remains exposed. Importantly, this band did not appear in a fresh mixture of QP and free cetuximab, indicating that a chemical bond between QP and cetuximab did occur in QPC synthesis, and this band is not a result of electrostatic reaction.

After confirming the chemical binding of cetuximab, verifying its inhibitory activity and ability to bind to the EGFR remained intact, was crucial. EGFR phosphorylation and the continuation of signals in the cascade was examined on CAL33 cells following 5 h of incubation with QPC in an aqueous solution and QPC^Cy7.5^ in a 5% dextrose solution as can be seen in Figure 1E. The inhibition of EGFR pathway of both carriers (QPC and QPC^Cy7.5^) was similar to the existing inhibition of free cetuximab. While the EGFR underwent phosphorylation, the downstream protein within the cascade, ERK, remained inactive after incubation with cetuximab alone. (Figure 1E). Summarizing the aforementioned findings indicate that we have successfully produced a positively charged starch carrier conjugated with the cetuximab antibody, while preserving its biological activity. It is important to note that reversing the order of the conjugation, where the cetuximab antibody was first attached to the PEG chains and then to the Q‐starch, did not work, as the inhibition activity of the cetuximab was not preserved.

### 2.2 Characterization and Stability of the Complexes

**Figure 2:**
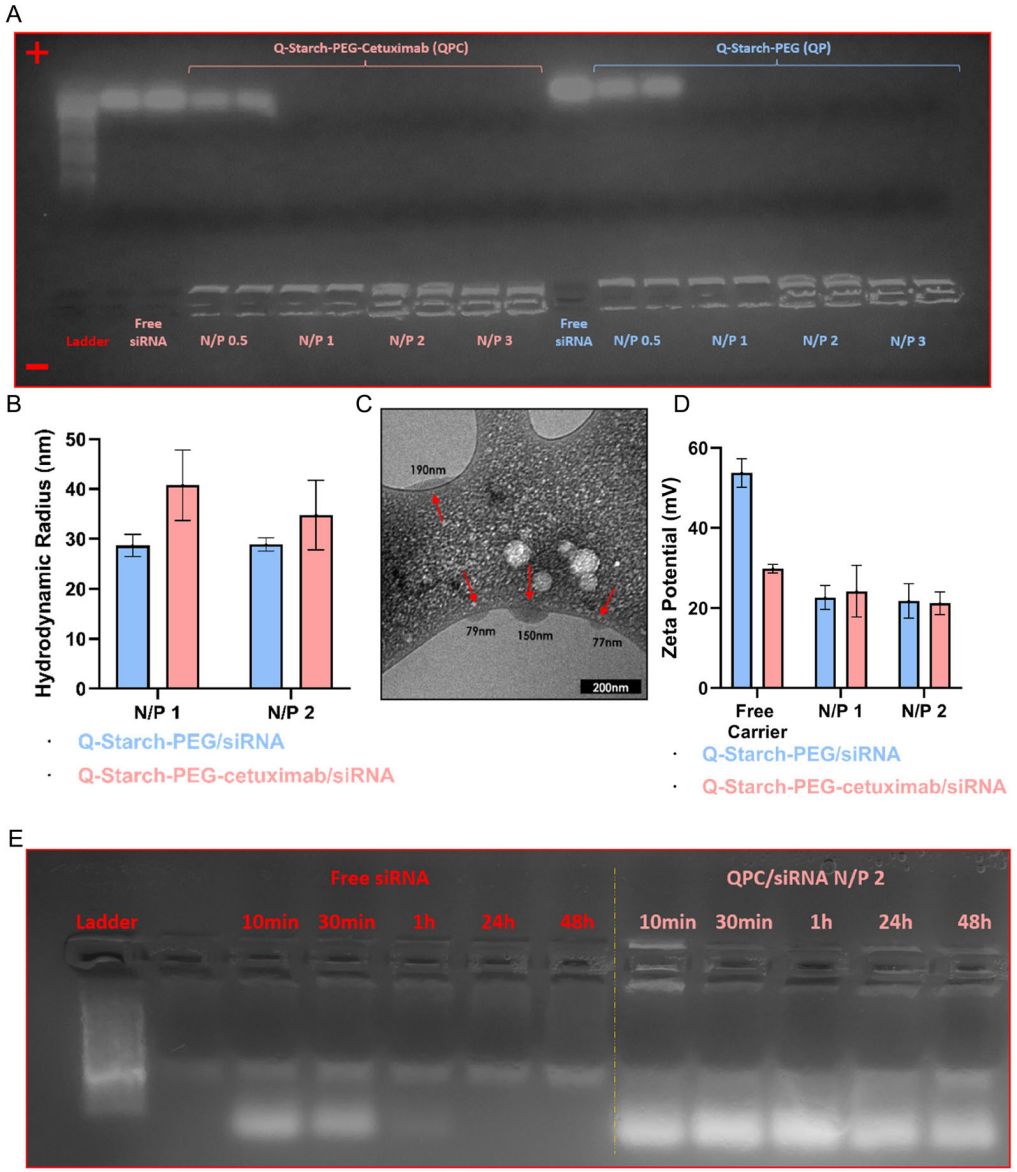
A) Q‐Starch‐PEG‐cetuximab (QPC)/siRNA and Q‐Starch‐PEG (QP)/siRNA complexes formation demonstrated by agarose gel electrophoresis (3% w/v) at different N/P ratios from 0.5 to 3. From the left, loading of a DNA ladder, free siRNA, and various N/P molar ratios of QPC/siRNA and QP/siRNA. B) DLS measurements of hydrodynamic radius of QP/siRNA and QPC/siRNA complexes at N/P ratios 1 and 2. (250 nM siRNA, mean ± SD (*n* = 3)). C) Representative cryo‐TEM images of QPC/siRNA complexes at an N/P ratio of 2 (siRNA at a concentration of 250 nM, Bar = 200 nm). D) Zeta potential (mV) of carriers alone, and QP/siRNA and QPC/siRNA complexes at N/P ratios of 1 and 2. (50 nM siRNA, mean ± SD (*n* = 3)). E) Gel electrophoresis results of stability test samples of free siRNA and QPC/siRNA complexes at N/P 2 (0.5 μg siRNA) at different incubation times in human serum, after which SDS (0.5% v/v) was added to de‐complex the siRNA from its carrier, allowing the run of naked siRNA.

The formation of QPC/siRNA and QP/siRNA complexes relies on electrostatic interactions between positively charged Q‐starch and negatively charged siRNA. The optimal N/P molar ratio, representing the ratio of positively charged amine groups in Q‐starch to negatively charged phosphate groups in siRNA, should be determined to ensure complete siRNA binding. The formation of QPC/siRNA and QP/siRNA at different N/P ratios was visualized using gel electrophoresis, and a representative result is depicted in **Figure** **2**A. As the N/P ratio increases from 0.5 to 3 for both, QPC/siRNA and QP/siRNA complexes, the intensity of the band in the bottom lane also increases. Upon addition of QPC or QP carrier to siRNA, the free fragments of siRNA interact with them through electrostatic interactions, thereby neutralizing the negative charges of siRNA (phosphate groups on siRNA backbone). Free siRNA migrates toward the positive electrode (anode) in the gel due to its negative charge (well observed for free siRNA and N/P ratio of 0.5). Starting from ratio 1, both carriers successfully entrapped siRNA molecules. Specifically, from ratio 2, the bands that did not migrate and remain in the well appeared darker, indicating a higher amount of siRNA in the complex.

The size, shape, and surface charge properties of nanoparticles are crucial when using them for the controlled release of drugs or genes, as these factors significantly affect their ability to overcome biological barriers. Complex's diameter size between 50 and 150 nm, is often suggested as the optimal size for this purpose.^[^ ^32^, ^33^ ^]^ This size range contributes to prolonged circulation time, reduces renal clearance, and enables effective penetration of to target tissues. As can be observed (Figure 2B), the average hydrodynamic radius of the QP/siRNA complex is 30 nm, and for QPC/siRNA complex, in the presence of cetuximab, is 35 nm at N/P molar ratio of 2. At the cryo‐TEM images of QPC/siRNA complex (Figure 2C, marked in red arrows), the complexes exhibited an elliptical shape rather than being perfectly spherical, and consequently, their calculated average size was therefore probably slightly affected. This deviation from sphericity can be attributed to the shear stresses applied during the sample preparation process for Cryo‐TEM analysis. In addition, the observed adherence of the complexes to the grid surface is likely due to electrostatic interactions between the positively charged complexes and the negatively charged carbon grid. Nevertheless, imaging of the complex is further evidence of the formation of a nanometric structure of our complexes.

Figure 2D presents the zeta potential of free carriers (QP and QPC), and complexes at N/P molar ratios 1 and 2. The carrier without the antibody exhibited a substantially higher surface charge compared to the carrier with the antibody. This observation suggests that the antibody may mask the carrier's charge. Conjugation of large antibody (152 kDa) can shield part of the Q‐starch backbone, which effectively reduces the surface charge. However, when the complexes were prepared, the surface charge of the complexes with the two different carriers remained similar. This can be explained by the spatial arrangement of the complex, where the antibodies probably are positioned away from the central core, preventing significant masking of the charge. The positive zeta potential values of the complexes, demonstrate the potential for cell internalization of our siRNA delivery system, since positive zeta potential can allow the complexes to penetrate through the negatively charged cellular membrane.^[^ ^34^ ^]^ In addition, these zeta potential values, around 20 mV, indicate that the complexes have moderate stability, with sufficient electrostatic repulsion to prevent significant aggregation. It is important to note that the presence of PEG also significantly contributes to the stability of the complexes by providing protection against serum proteins.^[^ ^35^ ^]^

Although no significant changes were observed in the complexes charge and size for both carriers, we chose to continue working with an N/P ratio of 2 from this point forward. To ensure that our complexes effectively reached their intended target organ through the targeting effect, which cetuximab antibody moiety adds to the polysaccharide carrier, examining the complexes’ stability in human serum was essential. As demonstrated in Figure 2E, siRNA in complexes form remains stable in serum for up to 48 h, in stark contrast to free siRNA, which degrades in less than an hour. These findings underscore the critical role of stability in the development of our delivery system, as it indicates that our complexes can circulate multiple times in the bloodstream, optimizing their opportunity to reach the target site effectively.

### 2.3 Complexes’ Cellular Uptake and Efficacy in Vitro

**Figure 3:**
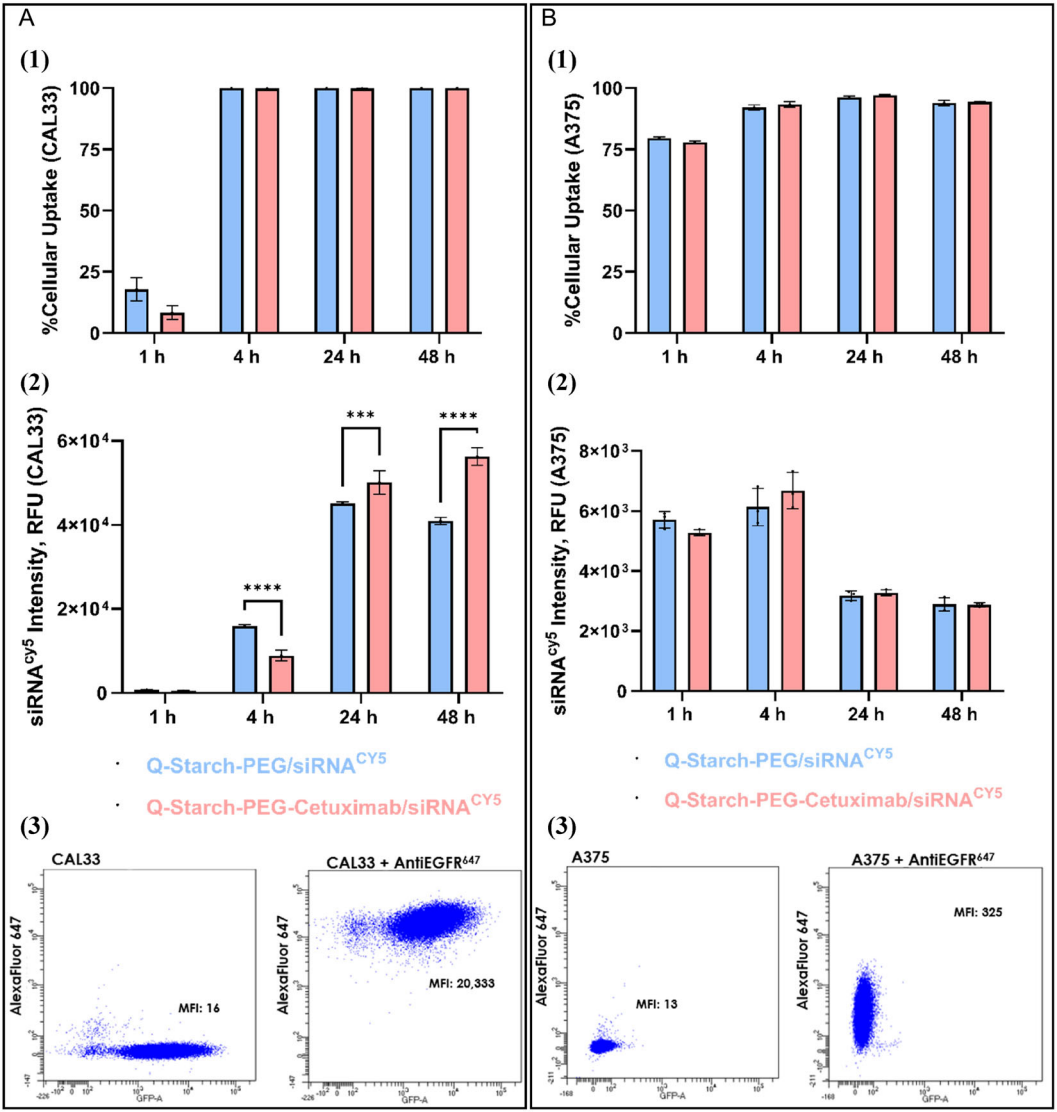
A) CAL33 (HNSCC cells) and B) A375 (melanoma cells). 1) %Cellular uptake and 2) Mean siRNA^Cy5^ intensity after 1 to 48 h of incubation with QP/siRNA (blue) and QPC/siRNA (pink) complexes at N/P 2. 50 nM siRNA, mean ± SD (*n* = 3), as assessed via FACSAria III. 3) FACSAria III analysis data of CAL33 and A375 cells pre‐treated with and without the Alexa Fluor 647 anti‐human EGFR antibody. Data presented at figures 1) and 2) assessed with two‐way ANOVA, *p < 0.05, **p < 0.01, ***p < 0.001, ****p < 0.0001.

The cellular uptake of QP/siRNA and QPC/siRNA by two cell lines; EGFR‐high expressing GFP‐CAL33 cells (**Figure** **3**A(3)), and EGFR‐low expressing A375 cells (Figure 3B(3)) were assessed via flow cytometry using Cy5‐labeled siRNA. The results, presented in Figure 3A(1),B(1), demonstrate that the entry of the complexes into A375 cells occurred faster, with ≈75% cellular uptake already after one hour (left panel) in contrast to the EGFR‐overexpressing CAL33 cells with an uptake of ≈20% (right panel). 100% of complexes cellular uptake was detected in CAL33 after 4 h, indicating that at least one complex has entered every cell. Upon evaluating the fluorescence intensity of the cells, which correlates with the number of complexes internalized by the cells, a slight advantage is observed for the complexes without the antibody after 4 h in CAL33 cells but not in A375 cell line (Figure 3A(2),B(2)). However, at longer time points, such as 24 h, this trend reverses, with the antibody‐containing complexes showing significantly higher levels of cellular uptake in CAL33 but no differences in A375. This advantage becomes even more pronounced after 48 h. We hypothesize that the difference in uptake rates indicate different entry mechanisms that occur due to antibody‐mediated receptor binding of QPC/siRNA compared to QP/siRNA complexes, leading to this increase in internalization over a longer period. Although ligand‐receptor internalization is typically fast ,^[^ ^36^ ^]^ it may be that when the antibody carries the complex, the kinetics changes. The conjugation of antibodies to nanoparticles may alter their internalization kinetics, often resulting in slower uptake compared to free antibodies. The intensity of the internalized siRNA molecules increases over time, suggesting continued spontaneous cellular uptake for both types of complexes. Interestingly, while CAL33 cells exhibited a gradual increase in fluorescence intensity over time, A375 melanoma cells showed a reduction in overall signal at 24 and 48 h. This phenomenon has been well‐documented^[^ ^37^ ^]^ in rapidly dividing cells such as melanoma, where the high proliferation rate results in dilution of intracellular fluorescence across daughter cells, thereby diminishing the overall signal even when the initial uptake is substantial.

**Figure 4:**
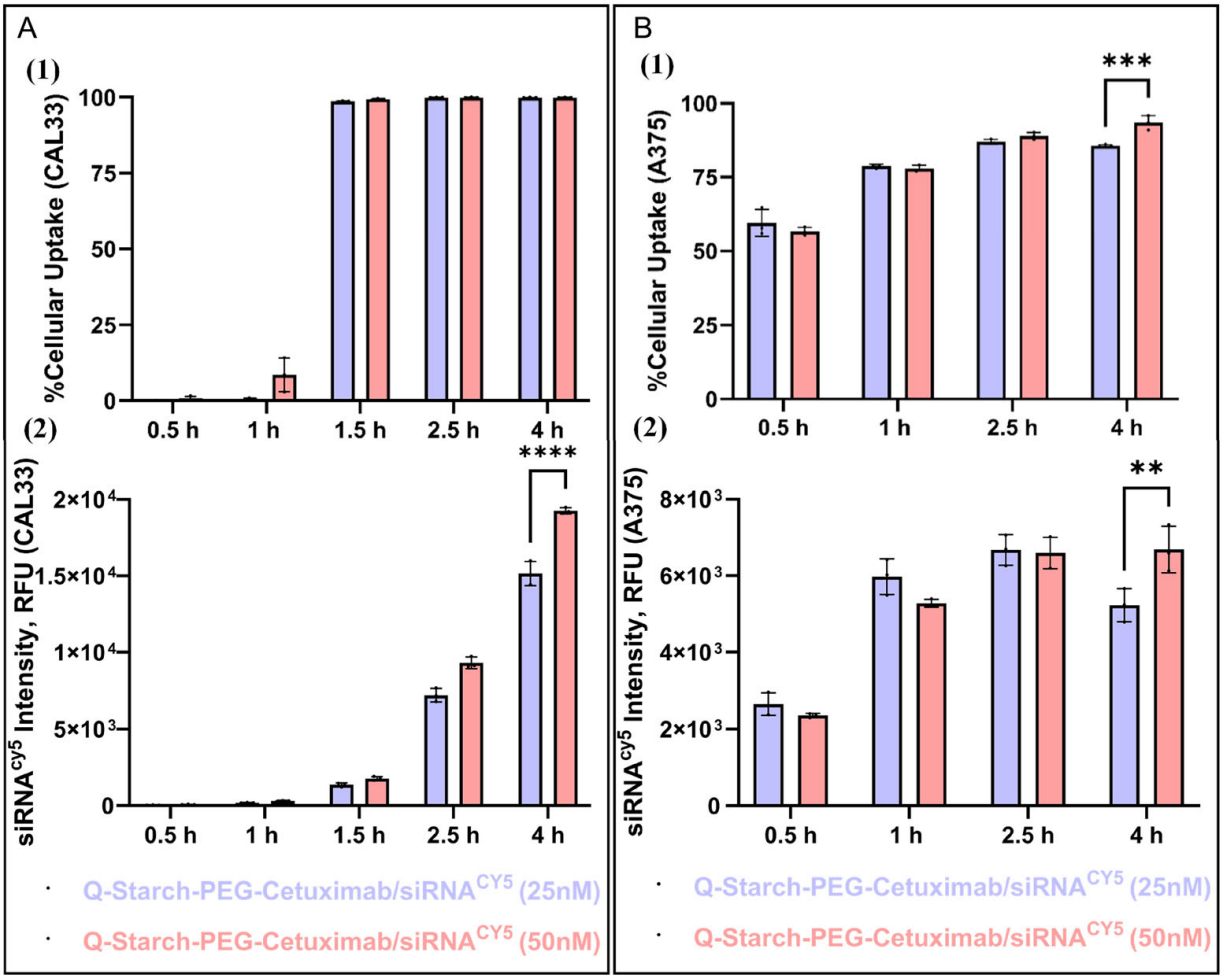
A) CAL33 (HNSCC cells) and B) A375 (melanoma cells). 1) %Cellular uptake and 2) Mean siRNA^Cy5^ intensity after 0.5 to 4 h of incubation with QPC/siRNA 25 nM (purple) and QPC/siRNA 50 nM (pink) complexes at N/P 2. Mean ± SD (*n* = 3), as assessed via FACSAria III. Assessed with two‐way ANOVA, *p < 0.05, **p < 0.01, ***p < 0.001, and ****p < 0.0001.

To evaluate the cellular uptake kinetics at the early time points after complexes’ incubation, QPC/siRNA^Cy5^ antibody‐conjugated complexes were incubated with CAL33 and A375 cells at two siRNA concentrations (25 and 50 nM) for up to 4 h. In CAL33 cells, a sharp increase in uptake percentage was observed between 1 and 1.5 h (**Figure** **4**A(1)), suggesting a defined onset of active internalization. Despite overall increase in fluorescence intensity over time, no significant differences were observed between concentrations during the first 2.5 h (Figure 4A(2)). A concentration‐dependent effect became apparent only at the 4‐hour mark, where the 50 nM group showed higher fluorescence than the 25 nM group, indicating a possible threshold‐dependent or rate‐limited mechanism.

Similarly, in A375 cells, no significant difference between the two concentrations was observed during the first 2.5 h. Only at the 4‐hour time point did a concentration‐dependent effect emerge, with the 50 nM group showing higher fluorescence intensity. These results suggest that in both cell types, initial uptake is not driven by siRNA concentration but becomes dose‐responsive with extended exposure time. The consistently rapid and steady uptake in A375 cells (Figure 4B(1),B(2)), contrasted to the more gradual uptake in CAL33 cells, supports our latest results (Figure 3), demonstrating that the complexes enter the cells through both spontaneous and receptor‐mediated pathways. In CAL33 cells, which overexpress EGFR, the delayed yet enhanced uptake aligns with receptor‐mediated endocytosis driven by cetuximab–EGFR interaction. Conversely, the efficient internalization observed in A375 cells, despite low EGFR expression, suggests that uptake occurs primarily through receptor‐independent mechanisms. This dual‐mode behavior highlights the versatility of our siRNA delivery system and the functional contribution of the antibody targeting over time.

**Figure 5:**
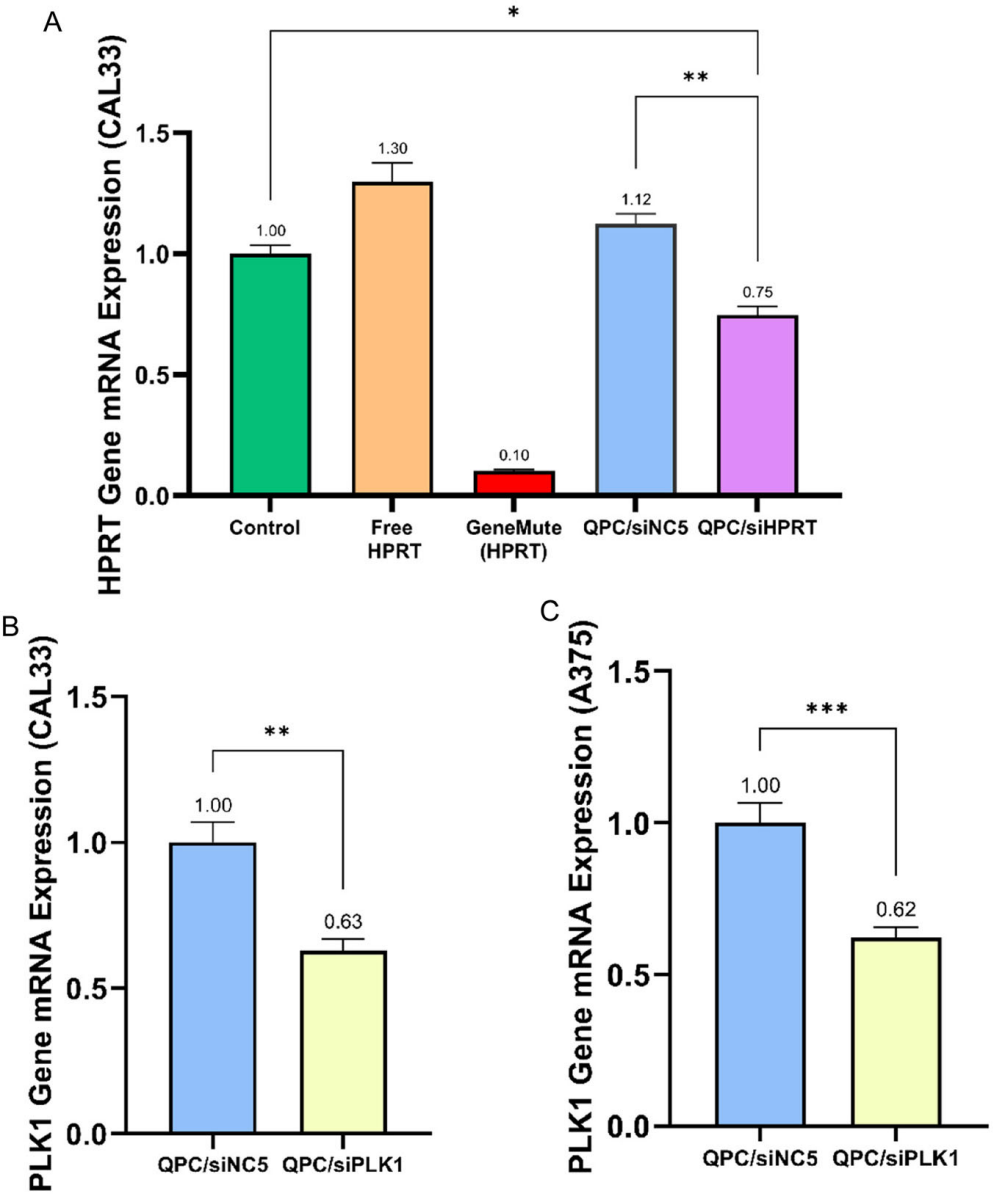
A) Gene silencing transfection with QPC/siHPRT and QPC/siNC5 complexes (utilized as off‐targeting control) following 72 h of incubation at N/P 2 in CAL33 cells. Additional controls included administration of free siHPRT and commercial transfection reagent GeneMute (50 nM). Gene silencing was quantified at the mRNA level using RT‐PCR and the results were normalized to the untreated cell, control (50 nM siRNA, mean ± SEM (*n* = 3)). B) Gene silencing transfection in CAL33 cells at the mRNA level using RT‐PCR following 72 h of incubation with QPC/siPLK1 complexes normalize to QPC/siNC5 complexes treatment (N/P 2, 50 nM siRNA, mean ± SEM (*n* = 3)). C) Gene silencing transfection in A375 cells at the mRNA level using RT‐PCR following 72 h of incubation with QPC/siPLK1 complexes normalize to QPC/siNC5 complexes treatment (N/P 2, 50 nM siRNA, mean ± SEM (*n* = 3)). (A) Assessed with two‐way ANOVA, (B and C) assessed with one‐way ANOVA, (D) assessed with t‐test. *p < 0.05, **p < 0.01, ***p < 0.001, and ****p < 0.0001.

Following the evaluation of complexes uptake kinetics across different concentrations, time points, and cell lines, we proceeded to examine their transfection efficiency. **Figure** **5**A presents gene silencing results in CAL33 cells, assessed via RT‐PCR. As shown, our complexes achieved 25% knockdown at the mRNA level when attempting to silence the Hypoxanthine‐guanine phosphoribosyltransferase 1 (HPRT1), serving as a model for gene silencing compared to the control. This decrease becomes even more significant, 33% knockdown, when normalized to complexes containing non‐coding siRNA (siNC5), suggesting potential off‐target effects caused by the entry of the complexes, specifically affecting this gene and leading to an increase in expression. Based on the results of administration of free siRNA, which did not induce knockdown in HPRT expression compared to the control, it can be concluded that the observed knockdown was facilitated using a carrier, which enabled the siRNA cell internalization and release into the cytoplasm, thus participating in the silencing mechanism. Although the transfection reagent (GenuMute) yielded impressive results with 90% silencing, it also caused a considerably high rate of apoptosis. In contrast, our complexes maintained high cell viability without inducing significant cell death. We investigated the silencing of an additional gene, PLK1, a promising target for cancer therapy due to its role in cell cycle regulation and its association with uncontrolled cell proliferation.^[^ ^38^ ^]^ Upon silencing PLK1, we observed consistent results, achieving ≈37% silencing compared to complexes containing non‐coding siRNA, QPC/siNC5 (Figure 5B). To validate this effect, we examined A375 melanoma cells which are also characterized by high PLK1 expression. As shown in Figure 5C, treatment with QPC/siPLK1 resulted in 38% knockdown, closely matching the effect observed in CAL33 cells. The reproducibility of gene silencing across two different genes and cell lines underscores the robustness of our siRNA delivery system and highlights its potential for broader therapeutic application across diverse genetic targets and tumor models.

While evaluating the efficacy of our system, it is important to consider the efficacy of alternative delivery methods reported in the literature. A recent review^[^ ^39^ ^]^ surveyed various methods including nanocarriers such as liposomes, polymeric nanoparticles, and exosomes, which improve the stability of siRNA, and prevent degradation and undesired bioaccumulation. They show that there is a range of gene therapy systems with gene knockdown efficiencies ranging from ≈35% to ≈90% at the mRNA level. As shown (Figure 5), we observed a 33%–38% knockdown at the mRNA level, which falls within the lower range of existing carriers.

Consistently, in the PLK1 silencing experiments performed in both CAL33 and A375 cells, no significant decrease in cell death was observed following treatment with QPC/siPLK1 complexes. This indicates that despite achieving 33%–38% knockdown at the mRNA level, the silencing was not sufficient to induce apoptosis. Given PLK1 known role in mitotic progression and survival, this outcome suggests that a more pronounced level of silencing may be required to induce a functional cytotoxic effect.^[^ ^40^, ^41^ ^]^ These findings reinforce the need for further optimization of the delivery system, particularly to enhance intracellular siRNA release and improve gene silencing efficacy.

Previously we presented detailed study^[^ ^10^ ^]^ evaluating the effect of the degree of substitution and starch molecular weight on the release kinetics. While decreasing the molecular weight of the starch enhanced siRNA release, the degree of substitution had no effect. However, the release of the siRNA remains within a certain boundary due to strong electrostatic interactions between the siRNA and Q‐starch carrier.^[^ ^42^ ^]^ The efficiency of the current carrier, QPC, which includes the cetuximab and the PEG, did not significantly improve upon the existing results.

### 2.4 Targeting Ability and Biodistribution of QPC/siRNA Complexes in Vivo

Rapid and disorganized angiogenesis in cancerous tumors leads to an increase in the permeability of the blood vessels surrounding the tumor,^[^ ^43^, ^44^ ^]^ resulting in enhanced permeation and retention (EPR) of molecules or particles up to 400 nm in diameter in the tumor compared to nearby tissues. This process enables the passive targeting of drugs to cancer cells. In this study, we employed passive and active targeting strategies to enhance the anti‐cancer therapeutic effect. Our results confirm that the targeted siRNA delivery system (QPC/siRNA complexes) is within the diameter size‐range that enables the EPR effect for passive targeting to the tumor in vivo. Moreover, our system was further modified to enable additional active targeting to cancer cells, by functionalizing the surface of the complexes with cetuximab, which recognizes EGFR. To evaluate which of these strategies control the accumulation of complexes in the tumor in vivo, we used knockdown EGFR cell line (shEGFR) and overexpressed EGFR tumor cell line (shControl). We generated these two cell lines by infecting CAL33 cells with viruses encoding either EGFR‐targeting sequences (shEGFR) or non‐targeting sequences (shControl). **Figure** **6**A illustrates three examples: shControl cells, which exhibit minimal fluorescence; shControl cells pre‐treated with the Alexa Fluor 647 anti‐human EGFR antibody, showing significantly higher fluorescence due to efficient binding to high level of EGFR; and shEGFR pre‐treated cells with the fluorescent antibody. The shEGFR sample demonstrates two distinct populations: **P1**, representing cells with low fluorescence intensity—sevenfold lower than the fluorescence observed in the **P2** population, which consists of cells with inefficient knockdown. The P1 population was enriched by FAC Sorter to obtain a purified cell population with low EGFR expression.

Figure 6A) FACSAria III sorting data of CAL33 cells (shControl cells, shControl cells pre‐treated with the Alexa Fluor 647 anti‐human EGFR antibody, and shEGFR pre‐treated cells with the fluorescent antibody), divided into two different cells populations according to their fluorescent antibody intensities. Zone P1 represents CAL33 cells with a low attachment of fluorescent antibody, and P2 represents CAL33 cells with a high attachment of fluorescent antibody. B) CAL33 shControl and shEGFR tumor growth curves, 1.5·10^6^ shControl cells/injection/tumor Vs. 4·10^6^ shEGFR cells/injection/tumor during 11 days of study (*n* = 5, mean ± SEM). Tumors volume was calculated by W^2^Lπ/6 (where: W = tumor width and L = tumor length). C) Schematic representation of the mouse experimental model. D, E): In vivo targeting of QPC^Cy7.5^/siRNA^Cy5^ complexes. Each column represents a different experimental group: the first column from left is a negative control mouse injected i.v. with 200 μL of 5% dextrose solution only. All the other columns represent mice that were injected i.v. with QPC^Cy7.5^/siRNA^Cy5^ complexes (10 μM siRNA, 200 μL per injection, *n* = 3). The upper rows of the images represent shControl tumors, and the bottom rows represent shEGFR tumors. The radiance of siRNA^Cy5^ ((D) Ex: 640, exposure: 5 sec) and QPC^Cy7.5^ ((E) Ex. 780 nm, exposure: 1.5 sec), were capture and analyzed by NEWTON 7.0 FT500 In Vivo Imaging 24 h post‐injection. Radiance, defined as the number of photons per second that leave a square centimeter of tissue and radiate into a solid angle of one steradian (sr), was quantified. The quantitative analysis of radiance for both siRNA^Cy5^ and QPC^Cy7.5^ is presented in D and E, respectively. (control *n* = 3, complexes *n* = 3, mean ± SEM). F) Quantitative analysis of fluorescence IVIS images, radiance of QPC^Cy7.5^ were obtained at the excitation wavelengths of 710 nm, with exposure times of 8 s normalized to tumors volume (*n* = 4, mean ± SEM). (E) Assessed with one‐way ANOVA, (F) assessed with t‐test. *p < 0.05, **p < 0.01, ***p < 0.001, and ****p < 0.0001.

**Figure:**
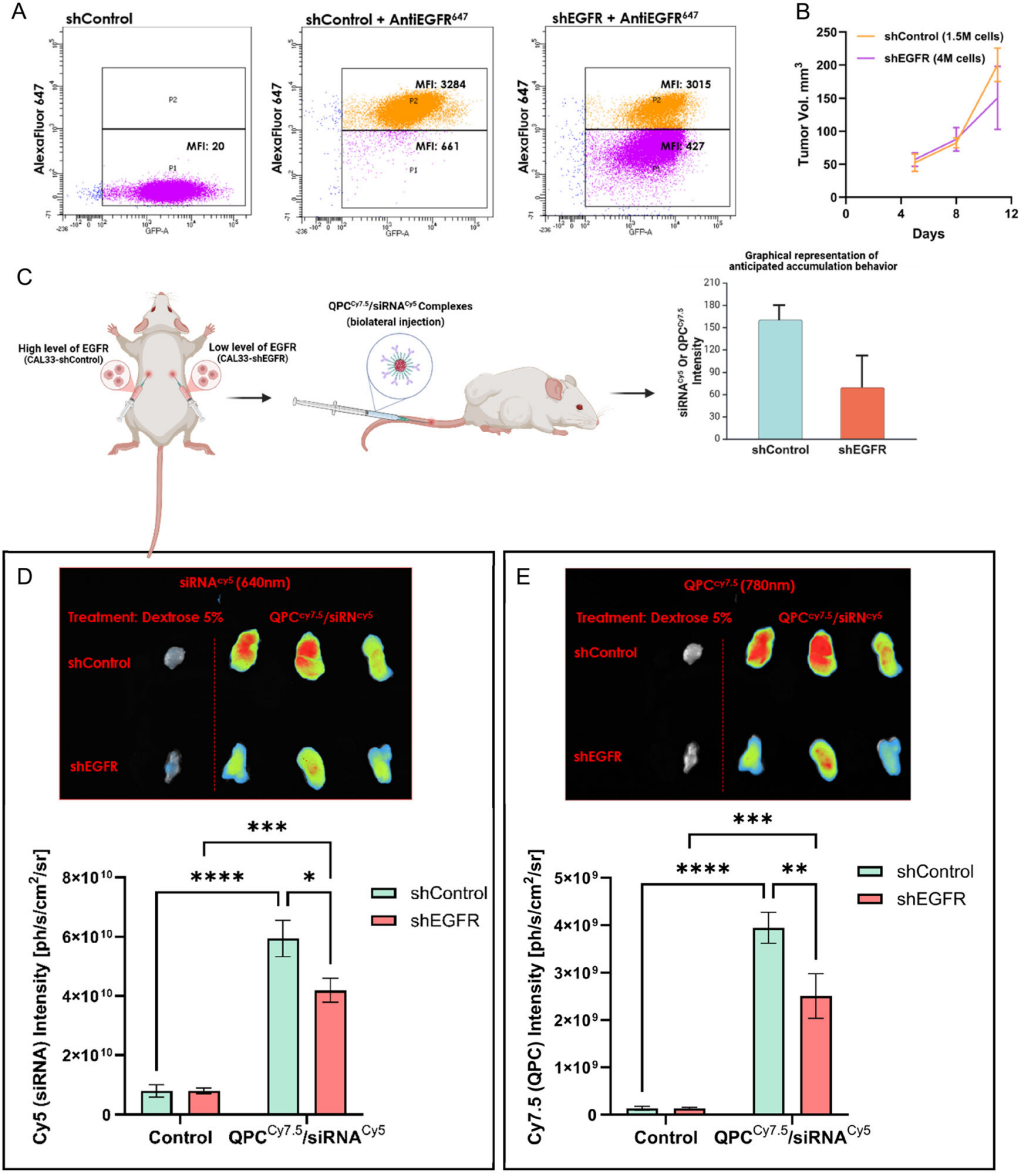

**Figure:**
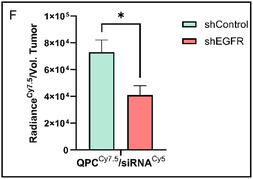

To establish tumors in mice, both shEGFR and shControl cell lines were used. Each mouse carried both types of tumors, serving as internal positive and negative controls. Preliminary experiments were conducted to determine the appropriate cell injection concentrations required to achieve tumors of comparable size. Different doses were tested by subcutaneous injection into two separate regions on the backs of NOD.Scid male mice. Tumor sizes were measured for 11 days after they became detectable, and their volumes (mm^3^) were calculated. As shown in Figure 6B, the optimized injection doses of 1.5 × 10^6^ cells for shControl tumors and 4 × 10^6^ cells for shEGFR tumors resulted in more consistent tumor growth. Following the establishment of the mouse model, we proceeded to evaluate whether there is a preferential active accumulation of the complexes in tumors exhibiting overexpression of the receptor (shControl), as compared to tumors with low receptor levels (shEGFR), following systemic administration via intravenous 24 h post injection (see Figure 6C). As shown in Figure 6D,E, there is a significant accumulation of complexes in both shControl and shEGFR tumors compared to the control mouse, which received a control injection of 5% dextrose without the complexes. This suggests that our complexes have inherent passive targeting capabilities to the tumor region via the associated vasculature, due to their physicochemical properties such as size and surface charge. Furthermore, both Cy5 (siRNA) and Cy7.5 (QPC) serve as effective fluorescent labeling molecules, as control tumors exhibit no auto fluorescent signal within their respective excitation/emission wavelength ranges. Notably, there is a higher accumulation of our complexes in shControl tumors compared to shEGFR tumors. This indicates that, in addition to the passive capabilities of the complexes to reach the tumor, they also possess superior active targeting abilities, as evidenced by the significantly higher accumulation in tumors with overexpress of EGFR (shControl). Moreover, the observation that both fluorescent labels showed similar accumulation indicates that the siRNA and the QPC carrier successfully arrived at the tumor as a complex rather than as separate entities. In a follow‐up experiment performed 48 h post‐injection, a similar pattern of accumulation was observed (Figure 6F). The complexes continued to show preferential localization in EGFR‐overexpressing tumors, further supporting the presence of a distinct kinetic profile and sustained stability of the QPC/siRNA complexes over time. Taken together, these findings highlight the strength of our delivery approach, which leverages both passive accumulation via the tumor vasculature and active targeting through EGFR recognition. Such dual functionality may improve therapeutic efficacy for specific tumor types while minimizing systemic clearance and enhancing pharmacokinetic performance.^[^ ^45^, ^46^ ^]^ This underscores the potential of our system as a versatile platform for targeted cancer therapy across heterogeneous tumor environment.

**Figure 7:**
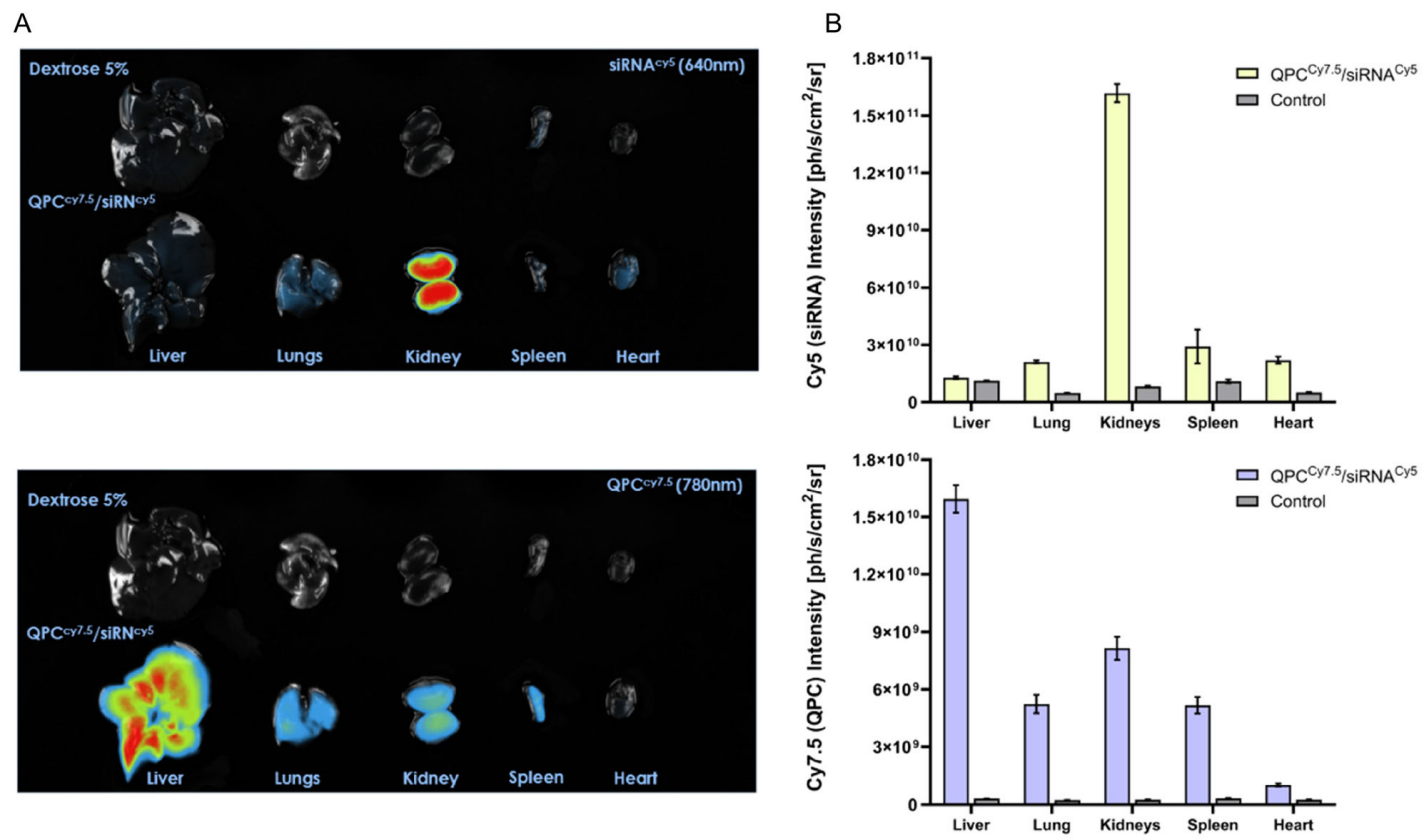
Biodistribution of QPC^Cy7.5^/siRNA^Cy5^ complexes in major organs 24 h post‐injection in vivo. A) The radiance of siRNA^Cy5^ (Ex: 640, 1.5 sec exposure time) and QPC^Cy7.5^ (Ex. 780 nm, 0.3 sec exposure time) were captured and analyzed by NEWTON 7.0 FT500 In Vivo Imaging 24 h post‐injection. B) Quantitative analysis results of radiance of siRNA^Cy5^ and QPC^Cy7.5^ were obtained at the respective excitation wavelengths of 640 nm and 780 nm, with exposure times of 1.5 s and 0.3 s. (control *n* = 3, complexes *n* = 3, mean ± SEM).

Since the QPC/siRNA complexes were given systemically, biodistribution studies also were done to evaluate the non‐specific uptake of the complexes by other essential organs in the body. We utilized the essential organs of the same mice which were treated by 5% dextrose solution (control, *n* = 3), and QPC^Cy7.5^/siRNA^Cy5^ complexes (*n* = 3). **Figures** **7**A,B present the fluorescent image of the essential organs (liver, lungs, kidney, heart, and spleen) and the quantitative analysis of tissue biodistribution data evaluated by two filters, 640 and 780 nm. Examination of the essential organs fluorescent intensity following injection of QPC^Cy7.5^/siRNA^Cy5^ complexes, revealed different intensity at each of the labeled component, QPC or siRNA. Direct comparison of the intensity levels between siRNA and QPC is not feasible due to the use of distinct fluorescent labels, Cy5 and Cy7.5, each requiring different exposure times; nevertheless, the resulting data can offer valuable insights into the temporal dynamics of complexes behavior. At 24 h post‐injection, we observed high accumulation of QPC at the liver, spleen, kidneys, and lungs, while the level of the siRNA is most significant in the kidneys. The observed rapid accumulation of free siRNA in the kidneys suggested renal clearance of siRNA known from the literature in mice.^[^ ^47^ ^]^ The rapid clearance of the positively charged carrier into the liver likely accrues since the complexes absorb serum proteins, such as serum albumin, which promotes their degradation over time and leads to clearance by resident macrophages of the mononuclear phagocyte system (MPS) in the liver, spleen, and lungs, resulting in their accumulation in these organs.^[^ ^48^ ^]^ The stability of our complexes was initially tested in vitro and was found to remain stable even after 48 h. Additionally, we observed a clear correlation between the accumulation of both fluorescent labels (siRNA and QPC carrier) in the tumors. This suggested that, while the complexes exhibit stability, there is also a natural degradation process occurring in the bloodstream. Complexes that do not accumulate in the tumors will eventually degrade and be eliminated by the body over time. The clearance of these complexes is crucial, as a safe delivery system must not only be effective but also capable of degrading and being eliminated from the body effectively. Further evidence supporting this accumulation trend is provided in the Supporting Information (Figure S1, Supporting Information), which include data from an extended group of five mice (*n* = 5).

## 3 Conclusion

In this study, we successfully targeted EGFR‐overexpressing tumors using a novel delivery system. In vivo experiments demonstrated that both siRNA and QPC carriers accumulated specifically in tumors with overexpression of EGFR compared to these with low EGFR expression within the same mouse, highlighting the system's selective targeting capabilities. Our findings demonstrate the potential of the system to protect siRNA over time and maintain its therapeutic, achieving 33%–38% knockdown of target mRNA (HPRT and PLK1).

Importantly, kinetic studies revealed distinct uptake dynamics between the different EGFR‐expressing cell lines, supporting a dual mechanism of passive and active targeting. However, the level of gene silencing achieved, while reproducible and biologically relevant, was not sufficient to induce apoptosis, indicating the need for further optimization to enhance intracellular release and silencing efficiency. We believe that the QPC starch‐based delivery platform holds strong potential for adaptation to a variety of therapeutic molecules, including DNA, mRNA, chemotherapeutic agents, and radiosensitizers. With the appropriate optimizations, particularly to improve controlled release, this system may become a versatile and effective tool for targeted cancer therapy.

## 4 Experimental Section

4.1

4.1.1

##### Materials

Soluble starch (S‐2630), sodium hydroxide (S‐0399), 3‐chloro‐2‐hydroxypropyl trimethyl ammonium chloride (CHPTAC, 348287), dialysis cellulose membrane (D9652), phosphate buffered saline (PBS, P4417), DMSO (dimethyl‐sulfoxide) (D2650), 1‐ethyl‐3‐(3 dimethylaminopropyl) carbodiimide (EDC·HCl, E6383), 4‐dimethylaminopyridine (DMAP, 107700), hydrochloric acid 32% (7647‐01), phosphatase inhibitor cocktails (P2714), anti‐MAP kinase (ERK‐1, ERK‐2) rabbit (M5670), gel loading solution (G2526), polybrene (H9268), dimethyl sulfoxide‐D6 (1034240050), and deuterium oxide (D_2_O, 113 366) were purchased from Sigma‐Aldrich (USA). Cetuximab (Erbitux, Merck), was kindly provided by Soroka Medical Center (Israel). TAE buffer 50X (20502375), acetone (01030521), and ethanol (05250502) were purchased from Bio‐Lab (Israel). The near‐infrared (NIR) fluorescent dye, cyanine7.5‐carboxylic acid (Cy7.5, 46090), was purchased from Lumiprobe Life Science Solutions (Germany). COOH‐PEG‐NHS 2 kDa (PG2‐CANS‐2 k) was purchased from Nanocs (USA). DMEM medium (01‐050‐1 A), fetal bovine serum (FBS, 04‐121‐1A), trypsin ethylenediaminetetraacetic acid (EDTA, 03‐052‐1B), L‐glutamine (03‐020‐1B), penicillin‐streptomycin (03‐031‐1B), trypan blue (03‐102‐1B) and the EZ‐PCR mycoplasma test kit (20‐700‐20) were purchased from Biological Industries (Israel). Phosphatase inhibitor cocktail (B15001) was purchased from Bimake (USA). Protein assay dye reagent concentrate (5 000 006), laemmli sample buffer (1 610 737), 0.2 μm PVDF transfer membrane (1 704 157), coomassie brilliant blue R‐250 (161–0436), 40% acrylamide/bis solution (161–0148), 1.5 M tris‐HCl buffer pH = 8.8 (161–0798), ammonium persulfate (161–0700), 0.5 M tris‐HCl buffer pH = 6.8 (161–0799), tetramethylethylenediamine (TEMED) (161–0801), tris/glycine/SDS buffer X10 (161–0772), tris/glycine buffer X10 (161–0734), and clarity western ECL substrate (170‐5060) were purchased from Bio‐Rad (USA). EGF receptor (D38B1), rabbit mAb (4267), phospho‐EGF receptor mouse mAb (2236), phospho‐p44/42 MAPK Erk1/2 rabbit mAb (4370), mouse (7076S), and rabbit (7074S) horseradish peroxidase (HRP)‐conjugated secondary antibodies were purchased from Cells Signaling Technology (USA). Alexa Fluor 647 anti‐human EGFR (BLG‐352918), and BSA (0216006980X) were purchased from ENCO (Israel). SeaKen LE agarose (50004) was purchased from Lonza (Switzerland). GenMute transfection buffer (SL100572), and PolyJet transfection reagent (SL100688) were purchased from SignaGen (USA). Presto Blue (A13262), and puromycin (A11138‐03) were purchased from Bioneer (USA). GeneRuler ultralow range DNA ladder (SM1211), StepOnePlus real‐time PCR system (4376600), TaqMan fast advanced master mix (4444557), and TaqMan gene expression assay (GAPDH: Hs99999905, HPRT: Hs99999909, PLK1: Hs00983227) were purchased from Thermo Fisher Scientific (USA). Ethidium bromide (E73298) was purchased from TAMAR (Israel). PureLinkTM RNA mini kit (12183020), and QuibitTM RNA BR assay kit (Q10210) were purchased from Invitrogen (USA). qScriptTM cDNA synthesis kit (95047‐100) was purchased from Quanta‐bio (USA). Plasmid purification jetstar maxi kit (220010) was purchased from Genomed (Austria). siRNA targeting against the human gene HPRT1 (51‐01‐08‐03), and noncoding siRNA (siNC5, 231781443) were purchased from Integrated DNA Technologies (IDT, USA). siNC5 sense: 5′‐CUAACGCGACUAUACGCGCAAUAUGGU‐3′ (Mw 16 500 g/mol), and custom non‐targeting siNC5 synthesis (5′‐Cy‐5 on sense strand purification for in‐vivo, HPLC) were purchased from Dharmacon (USA).

Sense: 5′Cy5.C.U.mA.A.C.G.C.G.mA.C.mU.A.mU.A.C.G.C.G.C.A.A.U.mA.U.mG.mG.mU3.

Antisense: 5′mC.mA.U.mA.U.mU.G.C.G.C.G.mU.A.mU.A.mG.U.mC.G.C.G.U.U.mA.G3′.

##### QPC Carrier Synthesis: Starch Quaternization (Q‐Starch Synthesis) and Labeling with Cy7.5

Quaternary starch (Q‐starch) synthesis and labeling with cyanine‐7.5‐carboxylic acid (Cy7.5) were carried out as described by Amar‐Lewis et al.^[^ ^14^ ^]^ Briefly, soluble potato starch (500 mg, 0.003 mol anhydrous‐glucose units) in sodium hydroxide solution (10 mL, 0.19 g Ml^−1^, 0.0475 mol) reacted with the CHMAC quaternization reagent (7.8 mL, 9 g, 0.0478 mol) for 24 h at room temperature (RT). The product was precipitated using an acidified mixture of ethanol and acetone (1:3 v/v) with 1 vol% HCl. The residue was washed gently with 80 vol% ethanol (100 mL), dissolved in a small volume (3‐4 mL) of deionized distilled water (DDW), and dialyzed against DDW for 48 h. The dialyzed product was then dried by lyophilization for 72 h. For Cy7.5 labeling, 231 mg of Q‐starch were dissolved in 13.0 mL of dimethyl sulfoxide (DMSO) solution. 15 mg of Cy7.5 were dissolved in 0.2 mL of DMSO, and added to the Q‐starch solution. Then, 111 mg of 1‐ethyl‐3‐(3‐dimethylamino propyl) carbodiimide (EDC) and 37 mg of 4‐dimethylamino pyridine (DMAP) were added to the above mixture, and the reaction mixture was stirred for 48 h at RT and kept covered with aluminum foil in a dark room. After 48 h, 4 mL of DDW were added to the above reaction mixture and poured into a 12 kDa cutoff dialysis bag. The labeled Q‐starch‐Cy7.5 was separated from the free (non‐reacted) Cy7.5 by extensive dialysis against PBS solution (pH 7.4, 5 PBS pellets were dissolved in 1000 mL of DDW) for 72 h, and then against DDW for additional 48 h, both in dark conditions. The dialyzed product was then freeze‐dried and lyophilized for 72 h to obtain the purified Q‐starch^Cy7.5^.

##### QPC Carrier Synthesis: Q‐Starch PEGylation (Q‐Starch‐PEG‐NHS Synthesis)

200 mg of Q‐starch or Q‐starch^Cy7.5^ and 30 mg of COOH‐PEG‐NHS (2 kDa) were dissolved in 12 mL of DMSO while stirring for 5 min at RT. Then, 60 mg of EDC and 20 mg DMAP were added to the above reaction mixture. The reaction mixture was stirred for 48 h at RT. Then, the solvent and unreacted substances were removed using 12 kDa cutoff dialysis bags against 5 L of DDW for 72 h. The DDW was replaced twice with fresh DDW during the dialysis process. The dialyzed product was then freeze‐dried and lyophilized for 72 h to obtain the purified Q‐starch‐PEG‐NHS or Q‐starch^Cy7.5^‐PEG‐NHS, respectively.

##### QPC Carrier Synthesis: QPC Synthesis

The attachment of cetuximab antibody to the PEGylated Q‐starch was done as follows: 1.79 mg of cetuximab antibody were dissolved in 35 mL of freshly prepared PBS solution (1 PBS pellet was dissolved in 25 mL of DDW), the pH of the above cetuximab antibody solution was adjusted to 8.2‐8.3 with 1 M NaOH solution and stirred for 30 min at RT. Then, 115 mg of PEGylated‐Q‐starch or PEGylated‐Q‐starch^Cy7.5^ were added to the aforementioned cetuximab antibody solution and stirred for 3.5 h at RT. The product was purified by 12 kDa cutoff dialysis bag against 5 L of DDW for 24 h. The DDW was replaced twice with fresh DDW during the dialysis process. The dialyzed product was then freeze‐dried and lyophilized for 72 h to obtain the purified QPC or Q‐starch^Cy7.5^‐PEG‐cetuximab (QPC^Cy7.5^), respectively.

##### Chemical Analysis of the Synthesis Products

Starch quaternization (Q‐starch) and PEGylated starch (Q‐Starch‐PEG‐NHS) syntheses were confirmed by FTIR, ^1^H NMR, and elemental analysis (EA). FTIR was obtained in a Thermo‐Nicolet FTIR spectrophotometer (Model‐Nicolet iS50 FTIR), and samples were prepared in the form of potassium bromide (KBr) pellets. ^1^H NMR spectra were recorded on a 500 MHz Brucker spectrometer using D_2_O as a solvent. Nitrogen content (N% weight) was measured by the elemental analysis method using a Thermo Scientific FLASH 2000 NC Analyzer.

The amount of the cetuximab antibody attached to the final product (QPC carrier) in each synthesis was measured using the Coomassie Blue protein assay. Briefly, equal quantities of the cetuximab antibody (0.5 μg) were resolved by sodium dodecyl SDS‐PAGE using a 10% polyacrylamide gel. After electrophoresis, the gel was carefully removed from the cassette and placed in a plastic container. Coomassie Blue staining solution (0.1% Coomassie Blue R‐250 in 40% methanol and 10% acetic acid) was added to the container, covering the gel completely, and allowed to stain for 1 h with gentle agitation. After staining, the excess staining solution was discarded, and the gel was washed overnight with a destaining solution composed of 40% methanol and 10% acetic acid with intermittent changes of the solution until the background was transparent. Finally, the gel was photographed using an appropriate imaging system to visualize the protein bands.

##### Cell Culture

CALL33 cells (head and neck squamous cell carcinoma, HNSCC cell‐line), which highly express EGFR, used as a cancer cell model. A375 cells (melanoma), which express low levels of EGFR, served as a control cancer cell model.

The cells were cultured in DMEM growth media containing 1% L‐glutamine, 1% penicillin‐streptomycin, and 10% fetal bovine serum (FBS) in a sterile incubator (model CB 170 CO_2_ incubator‐BINDER) at 5% CO_2_ and 37 °C.

##### Biological Activity of QPC Carrier

The biological activity of the cetuximab attached to the starch backbone was examined by inhibiting the phosphorylation cascade of EGFR and western blot assay. Cal33 cells (6·10^5^) were cultured in 100 mm^2^ plates (Corning tissue‐culture treated culture dishes) for 48 h to reach cells’ confluence of 50%. Then, the cells were incubated with different treatments (400 μL) for 5 h, as follow: 1) DDW or 5% dextrose solution (negative control); 2) 60 μg of free cetuximab in DDW or 5% dextrose solution; 3) QPC carrier in DDW and QPC^Cy7.5^ in 5% dextrose solution. The weights of each carrier were adjusted based on calculation for each synthesis using the Bradford assay, ensuring that the final amount of antibody in each sample was identical (containing 60 μg of cetuximab). After incubation, the cells were collected and lysed with lysis buffer. Bradford assay kit was used to determine protein concentration. 20 μg of total lysate were resolved on NuPAGE 10% Bis‐Tris gels and electrophoretically transferred to transfer membranes. Membranes were blocked for 1 h in 5% BSA in Tris‐buffered saline (TBS)‐Tween (0.1%), and then hybridized with primary antibodies in 5% BSA and 0.1% of Tween. Mouse and rabbit horseradish peroxidase (HRP)‐conjugated secondary antibodies (1:10 000) were diluted in 5% BSA in TBS‐Tween. Protein‐antibody complexes were detected by chemiluminescence with ECL (Westar Supernova, Cyanagen XLS3.0100 and Westar Nova 2.0 Cyanagen XLS071.0250), and images were captured with an Azure biosystems camera system.

##### Agarose Gel Electrophoresis for N/P Ratio Evaluation

The desired nitrogen (N) to phosphorus (P) ratio (N/P), needed for achieving total complexation of siRNA was evaluated using a gel electrophoresis assay. 3 μL of siRNA^NC5^ (20 μM stock solution), either in its free form or complexed with Q‐starch‐PEG‐NHS (QP) or Q‐starch‐PEG‐cetuximab (QPC) at the desired N/P ratio (between N/P 0.5 and 3), were combined with ×6 loading buffer and applied (24 μL) onto a 3% agarose gel containing 0.2 μg mL^−1^ ethidium bromide for siRNA staining. The gel was then placed in a horizontal electrophoresis apparatus (Wide Mini‐Sub cell GT, BioRad) filled with ×1 Tris‐acetate EDTA (TAE) buffer solution (prepared in our lab as a ×50 stock solution), subjected to an electric field (160 V) for 40 min, and subsequently visualized using UV illumination (Visible and Ultraviolet Transilluminator, DNR Bio‐Imaging Systems).

##### Carrier/siRNA Complexes’ Physical Characteristics

The physical properties of the QP/siRNA and QPC/siRNA complexes at N/P molar ratio of 1 and 2 in DDW solution were evaluated by several methods; Dynamic light scattering (DLS) was used to determine the complexes’ hydrodynamic size distribution. Samples with a final concentration of 250 nM siRNA were prepared (final vol of 200 μL) and spectra were collected using CGS‐3 (ALV, Langen, Germany) with a laser power of 20 mW at the He–Ne laser line (632.8 nm). Correlograms were generated by ALV/LSE 5003 correlator, collected at 90°, for 15 s, 10 times, at 25 °C, and fitted using the CONTIN program. Complex's size is reported as the mean ± SD (*n* = 3).

The complexes were also visualized for their morphology using cryo‐TEM (transmission electron microscopy). 2.5 μL drop of complexes solution (same as the DLS preparation) was applied on a copper grid, coated with a perforated lacey carbon 300 mesh (Ted Pella Inc., Redding, CA, USA) under controlled temperature (cryogenic temperature), and blotted with filter paper to form a thin liquid layer. The blotted samples were immediately and automatically plunged into liquid ethane at its freezing point (−183 °C) using Plunger (Leica EM GP). The specimens were transferred into liquid nitrogen for storage. Samples were analyzed using FEI Tecnai 12 G2 TEM at 120 kV with a Gatan cryo‐holder maintained at −180 °C. Images were recorded on a slow‐scan cooled charge‐coupled device camera (Gatan, Pleasanton, CA, USA) at low‐dose conditions to minimize electron‐beam radiation damage. The recording was carried out using the Digital Micrograph software package.

The surface charge of the complexes was assessed through zeta potential measurements. Samples (250 nM siRNA, 1 mL total vol) were transferred to U‐tube cuvettes (DTS106 °C, Malvern) for subsequent zeta potential measurements using Zetasizer (ZN‐NanoSizer, Malvern, England). Each sample was measured in automatic mode, at 25 °C, and the Smoluchowski model was employed to calculate the zeta potential. The zeta potential value for each sample is reported as the average of three runs, and the average value for each N/P ratio is presented as the mean ± SD (*n* = 3).

##### Complex Stability in Human Serum

The stability of complexes in human serum was assessed using gel electrophoresis. Donation of human serum were obtained from two unrelated volunteers in our lab. 15 μL of free siRNA or QPC/siRNA complexes at N/P 2 were mixed with fresh human serum to achieve a 50% serum (vol%) concentration and then incubated at 37 °C. Then, at specified time intervals (10 min, 30 min, 1, 24, and 48 h) for both free siRNA and complexes, the samples were removed and incubated at RT with 0.5% (% v/v) sodium dodecyl sulfate (SDS) for 15 min to disassemble the protected siRNA from the QPC carrier. Subsequently, the samples were loaded into agarose gel electrophoresis (3% w/v), where intact siRNA protected from degradation migrates as a visible band upon staining with ethidium bromide.

##### Flow Cytometry Analysis of EGFR Expression

To evaluate surface expression levels of EGFR, 1·10^6^ CAL33 and A375 cells were transferred into a 1.5 mL Eppendorf tube and centrifuged at 260 g in RT for 5 min. Each tube was re‐suspended with FACS buffer (1% FBS in PBS) containing an Alexa Fluor 647 anti‐human EGFR antibody for 30 min on ice in the dark (5 μL of antibody per million cells in 100 μL staining volume according to the manufacturer's procedure). Then, the cells were diluted with FACS buffer to get a final volume of 1 mL, centrifuged at 260 g in RT for 5 min, and washed twice with FACS buffer (1 mL) to remove antibody residues. For fluorescent measurement, the cells were re‐suspended in 1 mL FACS buffer and transferred into a FACS tube on ice. Anti‐human EGFR Antibody staining was measured by flow cytometry using FACSAria III.

##### In vitro *Cellular Uptake Using Flow Cytometry*

To evaluate cellular uptake of siRNA complexes, fluorescently labeled siNC5^Cy5^ (excitation at 633 nm) was complexed with the different carriers, and internalization was quantified using flow cytometry. CAL33 and A375 cells were seeded in a 6‐well plate at a density of ≈1 × 10^5^ cells per well in DMEM growth media, 24 h prior to treatment. On the day of transfection, the medium was replaced with 800 μL of fresh growth medium. QP/siNC5^Cy5^ and QPC/siNC5^Cy5^ complexes were freshly prepared at an N/P ratio of 2 (250 μM siRNA stock) as described above. Two uptake studies were performed: (1) Long‐term kinetic study (1–48 h): conducted with both QP and QPC complexes in CAL33 and A375 cells at a final siRNA concentration of 50 nM. (2) Short‐term kinetic study (0.5–4 h): conducted with QPC/siNC5^Cy5^ complexes only, at final siRNA concentrations of 25 nM and 50 nM. For all experiments, following incubation, cells were washed twice with PBS to remove free complexes. Adherent cells were detached using 300 μL Trypsin‐EDTA (10 min at 37 °C), neutralized with 1 mL growth medium, and pipetted gently to obtain a single‐cell suspension. Cells were transferred to sterile 1.5 mL tubes, centrifuged at 260 g for 5 min at room temperature, and resuspended in 500 μL of PBS containing 1% FBS. Samples were kept on ice and protected from light for up to 30 min prior to analysis. The fluorescence unit of the cells was measured by BD FACSAria III (Becton, Dickinson and Company, Franklin Lakes, New Jersey, USA) *n* = 3 for each experimental group. In order to gate the cell population that uptake the complex, Relative Fluorescent Unit (RFU) of the treated cells was compared to the autofluorescence RFU of the untreated cells (i.e., untreated cells were considered as 0% uptake). Data analysis was done using FACSDiva v8.3 software.

##### siRNA Transfection‐HPRT and PLK1 Gene Silencing

Housekeeping (HPRT) and Polo‐like kinase 1 (PLK1) gene silencing experiments were conducted by seeding CAL33 and A375 cells in a 6‐well plate for 24 h in DMEM growth media at a concentration of 1·10^5^ cells per well to reach a confluence of ≈60%–70%. On the day of transfection, QPC/siRNA complexes, either with non‐targeting siRNA (siNC5) or with siRNA targeting the HPRT gene (siHPRT) or PLK1 (siPLK1), were prepared at an N/P molar ratio of 2. Before transfection, cell media was replaced, and particles were placed on top of the cells at a final siRNA concentration of 50 nM and a final volume of 1 mL per well, and incubated at 37 °C and 5% CO^2^ for 72 h. Following incubation, the total RNA from the cells was extract and purify using the PureLinkTM kit according to the manufacturer's instructions. Total RNA was reverse transcribed using a qScript cDNA synthesis kit according to the manufacturer's instructions. Real‐time PCR was performed (StepOne Real‐Time PCR Systems) using TaqMan master mix with matching probes (HPRT and GAPDH). Analysis was performed with the StepOne Software version 2.3. Fold change was calculated by the ΔΔCt method.

##### Production of Low EGFR Expression Cal33 Cell Lines

HNSCC with low expression of EGFR cell line (shEGFR), was produced by lentiviruses infection. Lentiviruses were initially transfected with HEK293 cells, using PolyJet transfection reagent according to the manufacturer's protocol. The lentiviruses were created with the following viral plasmids: packaging plasmid (psPAX2), vesicular stomatitis virus G (VSVG), and two shRNAs including non‐targeting sequences (shControl) and silencing of EGFR expression shEGFR. Viruses were collected 48–72 h after transfection and used for cells infection. Cal33 cells were seeded in 6‐well plates (4·10^4^ cells well‐1 to reach 30% confluence) and infected with lentiviruses (1 mL of lentiviruses and 1 mL of growth media in each well) and incubate for 24 h. Then, the media was changed with selection media (media containing puromycin with high selectivity for uninfected cells), until all the uninfected cells died (around one week). After the transfection, cells with reduced EGFR expression were sorted by FACS. In order to verify the amount of EGFR expression on the cells, 1·10^6^ infect shEGFR and shControl cells were transferred into a 1.5 mL Eppendorf tube and centrifuged at 260 g in RT for 5 min. Each tube was re‐suspended with FACS buffer (1% FBS in PBS) containing an Alexa Fluor 647 anti‐human EGFR antibody for 30 min on ice in the dark (5 μL of antibody per million cells in 100 μL staining volume according to the manufacturer's procedure). Then, the cells were diluted with FACS buffer to get a final volume of 1 mL, centrifuged at 260 g in RT for 5 min, and washed twice with FACS buffer (1 mL) to remove antibody residues. For fluorescent measurement, the cells were re‐suspended in 1 mL FACS buffer and transferred into a FACS tube on ice. Anti‐human EGFR Antibody staining was measured by flow cytometry using FACSAria III. The cells with low intensity of Alexa Fluor 647 (low conjunct with AntiEGFR antibody, that is, shEGFR) were collected into a 5 mL tube with a rich medium (10% FBS and 2% pen strep antibiotic) and seed in a 6‐well plate for further use.

##### QPC/siRNA Complexes Targeting Ability and Biodistribution in vivo

Male Nod. SCID mice, 2 months old, were maintained and treated according to the institutional guidelines of the Ben‐Gurion University of the Negev. Animal experiments were approved by the Institutional Animal Care and Use Committee (No 012_b17099_33). Mice were housed in air‐filtered laminar flow cabinets with a 12‐h light/dark cycle, and food and water ad libitum. For tumor model in vivo, CAL33‐shControl (1.5·10^6^ cells 100 μL PBS solution^−1^ per injection) and cal33‐shEGFR (4·10^6^ cells 100 μL PBS solution^−1^ per injection) were injected subcutaneously into anesthetized Nod. SCID mice at two different regions on their back. Tumors sizes were measured with a digital caliper, and tumors volume were determined using the formula of length × width^2^ × (π/6). When the tumors volume had reached ≈200 mm^3^, up to two weeks, the mice were injected with label complexes at N/P molar ration 2 (2 mg siRNA Kg^−1^) intravenously via the tail vein (QPC^Cy7.5^/siRNA^Cy5^, 10 μM siRNA, 200 μL vol of injection). At 24 and 48 h post injection, mice were euthanized with CO_2_. Tumors and major organs (liver, kidneys, lung, spleen, and heart) were excised, washed with PBS, and imaged for radiance signal. At 24 h post‐injection the radiance of siRNA^Cy5^ (Ex: 640 nm) and QPC^Cy7.5^ (Ex. 780 nm) were captured and analyzed using NEWTON 7.0 FT500 In Vivo Imaging based on region of interest (ROI) quantification. At 48 h post‐injection, a separate cohort of mice was euthanized, and tumors were processed identically but imaged using a different in vivo imaging system (IVIS Lumina, 200 series, Xenogen Corporation, Alameda, CA), equipped with Cy7.5‐specific filters (Ex. = 710 nm, Em. = 745 nm). Fluorescent signals were quantified using the Living Image 4.0 software package (Caliper Life Sciences) based on region of interest (ROI) quantification.

##### Statistical Analysis

All data processing was conducted using GraphPad Prism v10.4.1 (GraphPad Software, CA, USA). Results are expressed as the mean ± SD or ± SEM, with the sample size (n) for each study specified in the figure legends. Pairwise comparisons between two groups were performed using the t‐test, one‐way analysis of variance (ANOVA) was applied for comparisons among more than two groups, and two‐way ANOVA was utilized to assess interactions between two independent variables. Statistical significance was determined using a threshold of P < 0.05, with the following designations: *p < 0.05, **p < 0.01, ***p < 0.001, and ****p < 0.0001.

## Conflict of Interest

The authors declare no conflict of interest.

## Author Contributions

**Chen Benafsha**: conceptualization (lead); data curation (lead); formal analysis (lead); investigation (lead); methodology (lead); project administration (lead); validation (lead); visualization (lead); writing—original draft preparation (lead). **Limor Cohen**: conceptualization (supporting); investigation (supporting); methodology (supporting); project administration (supporting). **Leah Shimonov**: investigation (supporting). **Riki Goldbart**: conceptualization (equal); methodology (equal); project administration (equal); visualization (equal); writing—review and editing (lead). **Tamar Traitel**: conceptualization (equal); methodology (equal); project administration (equal); visualization (equal); writing—review and editing (equal). **Eliz Amar‐Lewis**: conceptualization (supporting); investigation (supporting); methodology (supporting). **Ramesh Chintakunta**: investigation (supporting). **Manu Parasad**: investigation (supporting). **Uzi Hadad**: investigation (supporting). **Moshe Elkabets**: conceptualization (equal); funding acquisition (supporting); methodology (equal); project administration (equal); resources (supporting); supervision (equal); visualization (equal); writing—review and editing (equal). **Joseph Kost**: conceptualization (lead); funding acquisition (lead); methodology (lead); resources (lead); supervision (lead); visualization (supporting); writing—review and editing (equal).

## Supporting information

Supplementary Material

## Data Availability

The data that support the findings of this study are available from the corresponding author upon reasonable request.
