# Supplementary material for: Targeted siRNA Delivery Using Cetuximab‐Conjugated Starch for Epidermal Growth Factor Receptor‐Driven Head and Neck Squamous Cell Carcinoma
Source: Small Sci. 2025 May 15;5(7):2500073. doi: 10.1002/smsc.202500073 (PMC12257885; doi:10.1002/smsc.202500073)
Supplement: Supplementary file 1 — Supplementary Material [file SMSC-5-2500073-s001.pdf]

Supporting Information

Targeted siRNA delivery using cetuximab-conjugated starch for EGFR-driven head and neck squamous cell carcinoma

Chen Benafsha, Limor Cohen, Leah Shimonov, Riki Goldbart, Tamar Traitel, Eliz Amar-Lewis, Ramesh Chintakunta, Manu Parasad, Uzi Hadad, Moshe Elkabets, Joseph Kost**

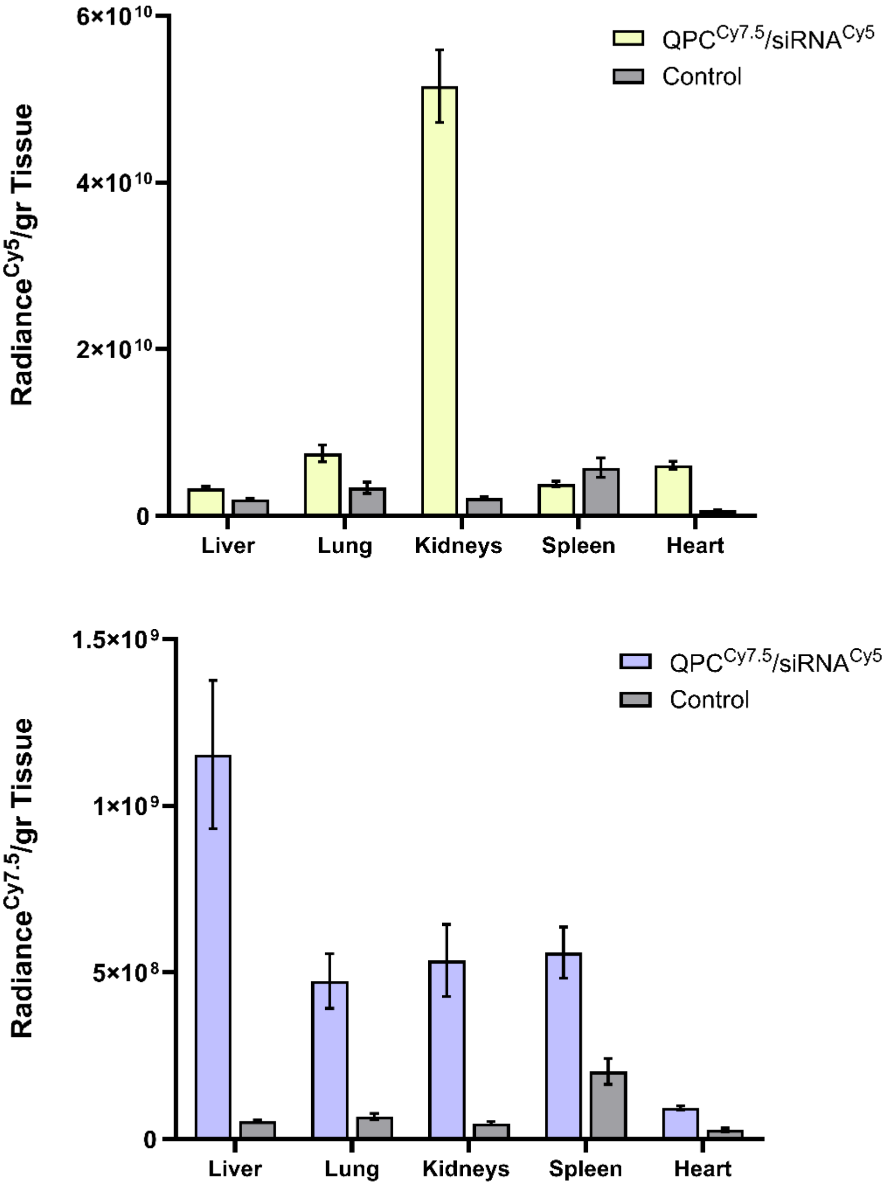

Figure S1: Quantitative analysis results of fluorescent images of major organs 24 hours post-injection in vivo. $siRNA^{Cy5}$ and $QPC^{Cy7.5}$ were obtained at the respective excitation wavelengths of 640 nm and 710 nm, with exposure times of 1 seconds for both. The radiance intensity is normalized to organ weight (n = 5, mean ± SEM).
